# 

**Published:** 1970-01

**Authors:** 


The
Bristol Medico-Chirurgical Journal
(Incorporating the Medical Journal of the South-West)
Established 1883

Vol. 85 (i) January 1970 No. 313

### BRISTOL MEDICO-CHIRURGICAL JOURNAL

Published Quarterly

Annual Subscription 16/- Post Free

### BRISTOL MEDICO-CHIRURGICAL SOCIETY

**President 1969-70: Dr. F. J. W. Lewis**
**President elect: Dr. Beryl Corner**
**Honorary Secretary: Dr. I. S. Bailey, 7 Percival Road, Bristol 8**
**Honorary Treasurer: Dr. Frank Ross, Bristol Royal Infirmary, Bristol 2**

The Society was founded in 1874. In 1883 publication of this Journal began and has continued quarterly since then. During the years 1949 to 1962 it appeared under the title of "The Medical Journal of the South West".

### THE BRISTOL MEDICO-CHIRURGICAL JOURNAL

**Editorial Committee**

| | |
|---|---|
| Editor | Dr. N. J. Brown, Southmead Hospital, Bristol |
| Assistant editor | Mr. J. B. M. Roberts |
| Members | Mr. H. B. Griffith<br>Dr. M. B. Lennard<br>Professor R. C. Wofinden<br>Dr. D. W. Wright<br>The Hon. Treasurer and Hon. Secretary of the Society |
| Business Manager | Dr. P. J. F. Baskett, Frenchay Hospital, Bristol |
| Secretary | Mr. A. E. S. Roberts, The Library, Medical School, University Walk, Bristol 8 |

### NOTICE TO CONTRIBUTORS

The Journal is especially concerned to provide a place for the recording of medical thought, interests, and practice in and around Bristol. It therefore welcomes articles that, as well as being of immediate interest to members, will document the local and contemporary medical scene for our successors.

Original articles are invited provided that they have not been published elsewhere. References in the text should be by author's name, with year of publication in parenthesis; they should be listed at the end in alphabetical order. Illustrations should be supplied ready for reproduction. Line drawings should be in Indian ink on firm white paper or board. The author will be informed if it is found necessary to ask him to pay for the making of blocks.

The following contributions will be especially welcome: notes of forthcoming events, meetings held, degrees conferred, staff appointments, honours and public appointments and other local news.

Twenty-five reprints of his article will be supplied free to each contributor. Quotations for larger quantities will be sent out with proofs and must be ordered when the corrected proofs are returned.

### The Medical Library

In 1890 The Bristol Medico-Chirurgical Society founded a medical library which in 1892 was combined with that of the Bristol Medical School. This became the University of Bristol Medical Library in 1925 and an agreement in that year confirmed the privilege of Members of the Society to use the University Medical Library.

### A Selection of Recent Additions to the Library

The Abortion Act 1967. Symposium by the Medical Protection Society (Pitman).
ADAMS, C. P. Dental photography. (Wright)
AMROMIN, G. D. Pathology of leukæmia. (Harper)
ARMSTRONG, M. L. Electrocardiograms. Ed. 2. (Wright)
BAIN, W. H. & HARPER, A. M. Blood flow through organs and tissues. (Livingstone)
BEAUMONT, G. E. Scientific and clinical medicine to-day. (Lloyd-Luke)
BELL, N. W. & VOGEL, E. F. eds. Modern introduction to the family. (Free Press, N.Y.)
BORDES, F. The old Stone Age. (World University Library)
BRITISH JOURNAL OF SURGERY, Great teachers of surgery in the past. (Wright)
BROTHWELL, D. R. & SANDISON, A. T. Diseases in antiquity. (Thomas)
BROTHWELL, D. R. The skeletal biology of earlier human populations. (Pergamon)
BUTLER, N. R. & ALBERMAN, E. D. eds. Perinatal problems (Livingstone)
BUTTERFIELD, W. J. H. Priorities in medicine. (Oxford U.P.)
CAILLET, R. Foot and ankle pain. (Davis)
CAINE, T. M. & SMAIL, D. J. Treatment of mental illness. (London U.P.)
CLARK, R. J. B. S., the life and work of J. B. S. Haldane. (Hodder & Stoughton)
CLARK, W. LeG. Chant of pleasant exploration. (Livingstone)
CLEAVE, T. L. & CAMPBELL, G. D. Diabetes, coronary thrombosis and the saccharine diseases. Ed. 2. (Wright)
CLELAND, W. et al Medical and surgical cardiology (Blackwell)
CLOSE, S. The know-how of infant feeding. Ed. 2 (Wright)
CLOSE, S. The know-how of pregnancy and labour. (Wright)
COOKE, R. E. Biologic basis of pædiatric practice. (McGraw-Hill)
COON, C. S. The origin of races. (Knopf)
COWIE, J. et al Delinquency in girls. (Heinemann)
CRADDOCK, F. W. Dental writing. (Wright)
DAVIES, I. J. T. Postgraduate medicine. (Lloyd Luke)
DUBOS, R. Man, medicine and environment. (Praeger)
DU PLESSIS, D. J. Principles of surgery. (Wright)
DUTHIE, J. J. R. & ALEXANDER, W. R. M. Rheumatic diseases. (Edinburgh U.P.)
EASTHAM, R. D. & JANCAR, J. Clinical pathology in mental retardation. (Wright)
EBLING, F. J. ed. Biology and ethics. (Academy Press)
ERIKSON, E. H. Identity: youth and crisis. (Faber)
EVANS, B. Caduceus in Saigon. (Hutchinson)
EXTON-SMITH, A. N. & SCOTT, D. L. eds. Vitamins in the elderly. (Wright)
FAMILY PLANNING for Ulster (Belfast Conference). (Wright)
FERRER, H. P. Screening for health. (Butterworth)
FISH, F. Outline of psychiatry. Ed. 2. (Wright)
FRASER, G. R. & FRIEDMAN, A. I. Causes of blindness in children. (Johns Hopkins Pr.)
FULFORD, G. E. & HALL, M. J. Amputation and prosthesis. (Wright)
GARDHAM, A. J. & DAVIES, D. R. Operations of surgery. Vol. 2. (Churchill)
GARDNER, L. I. ed. Endocrine and genetic diseases of childhood. (Saunders)
GIBLETT, E. R. Genetic markers in human blood. (Blackwell)
GOLIGHER, J. C. et al Ulcerative colitis. (Bailliere)
GUNZBERG, H. C. Social competence and mental handicap. (Bailliere)
HOLLINGSWORTH, J. W. Local and systemic complications of rheumatoid arthritis. (Saunders). Presented by Professor Perry
HOLLOWAY, P. J. et al. Child dental health. (Wright)
HOWELLS, W. Ideas on human evolution. (Harvard Univ. P.)
HSIA, D. Y. Human developmental genetics. (Yearbook Med. Publishers)
HUMAN VARIATION and origins. Readings from Scientific American. (Freeman)
JACKSON, C. R. S. The eye in general practice. Ed. 5. (Williams & Wilkins)
KAZNER, E. et al Proceedings in echo-encephalography. (Wright)
KEATINGE, W. R. Survival in cold water. (Blackwell)
KIRMIZ, J. P. Adaptation to desert environment (Butterworths)
KRASNOFF, S. O. Computers in medicine. (Thomas)
KURTEN, B. The age of the dinosaurs. (World University Library)
LANGSLEY, D. G. & KAPLAN, D. M. Treatment of families in crises. (Grune)
LIDZ, T. The person. (Basic Books)
MAINGOT, R. ed. Abdominal operations. 2 vols. Ed. 5 (Butterworths)
MEADE, R. H. Introduction to the history of general surgery. (Saunders)

OAKLEY, K. P. Framework for dating fossil man. (Weidenfeld & Nicolson)

PRYSE-PHILLIPS, W. Epilepsy. (Wright)

PULEC, J. L. Menière's disease. (Saunders)

RAY, G. E. Precision attachments. (Wright)

RECENT ADVANCES IN BLOOD COAGULATION. Edited by L. Poller. (Churchill)

ROBERTS, F. P. Breast feeding. (Wright)

ROSE, J. ed. Computers in medicine. (Churchill)

ROSENTHAL, D. & KETY, S. S. eds. The transmission of schizophrenia. (Pergamon)

ROYAL COLLEGE OF PHYSICIANS. Fifth symposium on advanced medicine. (Pitman)

RYCROFT, C. Anxiety and neurosis. (Lane)

SALING, E. Fœtal and neonatal hypoxia. (Arnold)

SCHEUER, P. J. Liver biopsy interpretation. (Bailliere)

SENN, M. J. E. & SOLNIT, A. J. Problems in child behavior and development. (Lea & Febiger)

SHELDON, J. M. et al Manual of clinical allergy. Ed. 2. (Saunders)

STRAUSS, M. B. Familiar medical quotations. (Churchill)

THOMAS, A. et al Temperament and behavior disorders in children. (London U.P.)

TRUMMER, M. J. & BERG, P. Lung transplantation. (Thomas)

TULLEY, W. J. & CRYER, B. S. Orthodontic treatment for the adult. (Wright)

TURK, J. L. Immunology in clinical medicine. (Heinemann)

WILSON, C. W. M. ed. Adolescent drug dependence (Pergamon)

WOLFF, S. Children under stress. (Penguin)

### Bristol Medico-Chirurgical Society

The Annual General Meeting was held in the Medical School on 8th October, 1969.

The President, Mr. A. L. Eyre-Brook resigned the Chair to his successor, Dr. F. J. W. Lewis who delivered his presidential address on "The History of Leukæmia" (to be published in a later issue of the Journal).

The following officers were elected:
President-elect: Dr. Beryl Corner.
Hon. Secretary: Dr. I. S. Bailey.
Hon. Treasurer: Dr. Frank Ross.

*The retiring treasurer Dr. J. Macrae was thanked by Dr. Alan Roberts who mentioned that in the last 43 years the Society had had only three treasurers, Dr. Macrae having held the office for no less than 16 years.*

Hon. Medical Librarian: Dr. G. E. F. Sutton.

Representatives to University Medical Library Sub-committee: Dr. M. Lennard, Prof. A. E. Read.

Three new members of Committee: Dr. J. A. Cosh, Mr. H. Espiner, Dr. K. Mallen. (The other members of committee are Mr. C. A. Brown, Dr. J. C. Causton, Mr. Huw Griffith, Dr. K. W. Miller, Dr. A. J. Rowland, Dr. P. S. Sinclair and Dr. J. E. Cates.)

Annual Reports were presented by the Hon. Secretary, Hon. Treasurer and Hon. Editor of the Journal. Dr. A. B. Raper, retiring Joint Editor, was warmly thanked for his services. Mr. Michael Roberts has been appointed Hon. Assistant Editor.

The present **Honorary Members** of the Society are Mr. A. Wilfred Adams, Dr. MacDonald Critchley, Dr. H. J. Orr-Ewing, Mr. Norman Tanner, Sir Philip Morris, Mr. H. J. Drew Smythe, Dr. G. E. F. Sutton, Dr. E. G. Bradbeer, Dr. A. G. Heron and Dr. Victoria Tyron.

### Programme for 1969-70

| | |
|---|---|
| 12th November: | Clinical Meeting, Bristol Royal Infirmary. |
| 10th December: | Dr. J. L. Emery: "Unexpected Death in Children". |
| 14th January: | Symposium on "Strokes" arranged by Dr. R. Langton Hewer. |
| 11th February: | Prof. J. Butterfield: "Priorities in Medicine". |
| 11th March: | Symposium on Medical Postgraduate Education in Bristol (Dr. J. E. Cates, Prof. A. E. Read, Mr. Huw Griffith and Dr. D. Ractliffe). |
| 8th April: | Symposium on Immunology arranged by Dr. J. Verrier Jones. |
| 15th May **(Friday):** | Sir Vivian Fuchs: "Human Endurance". |

### Postgraduate Education

**Sunday Morning Meetings at Southmead Hospital, 10.30 a.m.**

**Provisional Programme :**

| | |
|---|---|
| Jan. 18th, 1970 | Recent Advances |
| Jan. 25th : | Chronic Psychiatric Disease (a) in Childhood |
| Feb. 1st : | Chronic Psychiatric Disease (b) in the Adult |
| Feb. 8th : | Clinical Session |
| Feb. 15th : | Urinary Tract Infections |
| Feb. 22nd : | Dermatology Session, with special reference to the prevention of Chronic Skin Disease |
| Mar. 1st : | Paediatric Session, again with special reference to the prevention of Chronic Disease |
| Mar. 8th : | The Physician and Palliation |
| Mar. 15th : | The Surgeon and Palliation |
| Mar. 22nd : | General Practitioner Session : Case Presentation and Research |

### Notes and News

**Obituary:** Percy Phillips
Duncan Wood
Winifred Brinckman
D. Howard Davies
Charles E. Bartlett

Dr. A. E. Read, M.D., F.R.C.P., has been appointed to the Chair of Medicine, and Mr. J. H. Peacock, M.B., Ch.M., F.R.C.S., to a Chair in Surgical Science in the University of Bristol.

Dr. MacDonald Critchley has been re-appointed President of the World Federation of Neurology for a further period of four years.

Mr. R. V. Cooke has been elected a Vice-President of the Royal College of Surgeons of England.

Dr. J. Apley has been awarded jointly the Dawson-Williams Prize of the British Medical Association for "outstanding work in pædiatrics".

Dr. G. H. Tovey has been elected to the Council of the (now Royal) College of Pathologists.

F.R.C.P. (London): J. H. S. Heller

F. J. Y. Wood

M. E. Disney

June K. Lloyd

F.C.Path: A. C. Hunt

### UNIVERSITY OF BRISTOL

### Examination Results

M.D.: T. O. Dada, E. P. Hamblett, Barbara F. Smith.

Ph.D.: A. G. Hannam, M. Philpott, G. C. B. Randall, E. M. Rodriguez, J. W. Boyd, M. M. Rweyemamu.

D.P.H.: S. Ahmad, D. Bevan, A. M. George, M. Harrison, K. J. Kimmance, A. McCann, D. F. J. Malins, P. D. Matthews, D. W. Maxa, J. P. M. Paget, M. R. F. Reynolds, M. El Din El Sheikh El Tayeb, A. A. Tejuoso, M. J. Whitfield.

M.B. Ch.B.: Pass with Honours—M. Brock (with distinction in Surgery), A. V. Buxton, P. Hollingworth (with distinction in Obstetrics and Gynæcology), D. J. Howard (with distinction in Surgery.

Pass—R. B. Adkins (with distinction in Public Health), B. B. Azzan, A. O. Bada, N. E. Baldock, M. J. Boyce, M. C. Bray, P. Brown (with distinction in Public Health), N. H. Coward, R. G. Charles, P. J. Cook (with distinction in Public Health), J. E. Davidson, P. Davis, Ruth E. Day, P. R. Densham, J. B. Dodoo, Jean B. Doole, A. Dosumu, Andrea M. Eastman, A. Fishtal, R. N. Ford, Janet E. Foster, T. C. Franklin, G. R. Genovese, P. Garner, D. M. Goodall, Diane E. P. Gurd, F. J. Hacking, J. M. Hammond-Evans, M. S. Hamza, R. D. Harding, K. Holden, Elizabeth T. Houang, Angela C. Knights, N. M. Moaby, R. Maraj, P. A. R. Morley, P. G. Murdin, F. Nahai, A. G. Negus, R. A. Payne, B. Pettit, R. G. Parks, D. E. Pickersgill, R. C. Preston, S. Price, A. B. Roberts, K. Rogers, J. N. Sell, F. C. Smith, N. C. W. Smith, G. L. Steer, P. Stillman, R. H. Teague, N. O. T. Temple, H. G. Thompson, S. J. Tovey, Julia M. Van Senten, G. J. Walsh, T. Ward, R. Windle, J. W. Witcher.

### Recent Consultant Appointments in the Bristol Clinical Area

**I. D. Chisholm, M.A., M.B., M.R.C.P., D.P.M.** Consultant Psychiatrist, Glenside and Barrow Hospitals.

**K. Joy Harrison, M.D., M.C.Path., D.C.H.** Consultant Pathologist, Frenchay Hospital.

**J. B. Parsons, M.B., F.F.A.R.C.S.** Consultant Anæsthetist, Weston-super-Mare.

**R. W. Piggott, M.B., F.R.C.S., F.R.C.S.I.** Consultant Plastic Surgeon, Frenchay Hospital.

**A. M. Kingon, M.B.E., M.B., M.R.C.P.** Consultant Geriatrician (with interest in General Medicine), Weston-super-Mare.

**R. Greenbaum, M.B., F.F.A.R.C.S.** Consultant Anæsthetist, Cossham-Frenchay Group.

**I. D. Fraser, M.D., M.C.Path.** Consultant Pathologist, Regional Transfusion Centre, Bristol.

**M. J. Gay, M.B., D.C.H., D.P.M.** Consultant Child Psychiatrist, Bristol.

**M. Nicholas, M.B., D.P.M.** Consultant Psychiatrist (psycho-geriatrics), Bristol.

**J. C. Mackenzie, B.A., M.B., M.R.C.P.E., D.I.H.** Consultant Nephrologist, Southmead and Ham Green Hospitals and U.B.H.

**G. R. Burston, M.B., M.R.C.P.E.** Consultant Geriatrician, Bristol.

**A. L. Hilton, B.A., M.B., M.R.C.P.** Consultant V.D., Bristol and Bath.

**Ruth M. Walters, M.B., D.C.H.** Consultant Psychiatrist (subnormality), Stoke Park Group.

**J. L. E. W. Thomas, M.B., F.F.A.R.C.S., D.T.M. & H.** Consultant Anæsthetist, U.B.H.

**W. K. Eltringham, M.B., F.R.C.S.** Consultant Surgeon, U.B.H.

### BURDEN RESEARCH MEDAL AND PRIZE

The Burden Trust has founded an annual award to be known as "The Burden Research Medal and Prize" to commemorate the Diamond Jubilee of the foundation of Stoke Park Hospital by the Rev. H. N. Burden on 1st April, 1909, and to encourage future research in the field of mental subnormality.

The Burden Research Medal and Prize for 1969, total value £250, may be presented on or about 1st April, 1970, at Stoke Park Hospital, for outstanding research work which has been published or presented as a paper to a learned society during 1969.

The award is open to all registered medical practitioners who are working in the field of mental subnormality in the United Kingdom or Republic of Ireland.

Further information is available from the secretary, Burden Trust, 16, Orchard Street, Bristol, 1.

